# Finding a Needle in a Haystack: The Role of Electrostatics in Target Lipid Recognition by PH Domains

**DOI:** 10.1371/journal.pcbi.1002617

**Published:** 2012-07-26

**Authors:** Craig N. Lumb, Mark S. P. Sansom

**Affiliations:** Department of Biochemistry, University of Oxford, Oxford, United Kingdom; University of Houston, United States of America

## Abstract

Interactions between protein domains and lipid molecules play key roles in controlling cell membrane signalling and trafficking. The pleckstrin homology (PH) domain is one of the most widespread, binding specifically to phosphatidylinositol phosphates (PIPs) in cell membranes. PH domains must locate specific PIPs in the presence of a background of approximately 20% anionic lipids within the cytoplasmic leaflet of the plasma membrane. We investigate the mechanism of such recognition *via* a multiscale procedure combining Brownian dynamics (BD) and molecular dynamics (MD) simulations of the GRP1 PH domain interacting with phosphatidylinositol (3,4,5)-trisphosphate (PI(3,4,5)P_3_). The interaction of GRP1-PH with PI(3,4,5)P_3_ in a zwitterionic bilayer is compared with the interaction in bilayers containing different levels of anionic ‘decoy’ lipids. BD simulations reveal both translational and orientational electrostatic steering of the PH domain towards the PI(3,4,5)P_3_-containing anionic bilayer surface. There is a payoff between non-PIP anionic lipids attracting the PH domain to the bilayer surface in a favourable orientation and their role as ‘decoys’, disrupting the interaction of GRP1-PH with the PI(3,4,5)P_3_ molecule. Significantly, approximately 20% anionic lipid in the cytoplasmic leaflet of the bilayer is nearly optimal to both enhance orientational steering and to localise GRP1-PH proximal to the surface of the membrane without sacrificing its ability to locate PI(3,4,5)P_3_ within the bilayer plane. Subsequent MD simulations reveal binding to PI(3,4,5)P_3_, forming protein-phosphate contacts comparable to those in X-ray structures. These studies demonstrate a computational framework which addresses lipid recognition within a cell membrane environment, offering a link between structural and cell biological characterisation.

## Author Summary

Cell signalling pathways are crucial for many biological processes including cell proliferation and survival. Signalling is governed by a complex network of interactions within the cell, and disruption of signalling can lead to a variety of human diseases. Often, a key event in the signalling cascade is the reversible recruitment of peripheral membrane proteins to the surface of the cell membrane, where they then bind to a specific lipid in order to perform their function. However, it is not clear how these proteins locate their target lipid in the complex multi-lipid environment of the plasma membrane. Here, we have used a combination of computational techniques to simulate the association of a signalling protein with the surface of the cell membrane. We demonstrate that the mechanism of membrane binding is dependent upon the lipid composition of the lipid bilayer, and the results show that orientational and positional steering of the protein is optimised when the anionic lipid content of our model membrane matches the physiological composition observed in cells.

## Introduction

The association of peripheral proteins with the cytoplasmic leaflet of the plasma membrane is an important step in a diverse array of cellular processes, from cell signalling to membrane trafficking [1]. The cytoplasmic leaflet of the eukaryotic cell membrane carries a net negative surface charge owing to the presence of anionic lipids [2], and recruitment of cytosolic proteins to the membrane is often achieved with the aid of these lipids [3]. Anionic lipids are thought to constitute between 10–15% [1], [4], [5], [6], [7] of the total lipids in the plasma membrane, and to be largely present in the inner (*i.e.* cytoplasmic) leaflet of the bilayer. The bulk of these anionic lipids, for example phosphatidylserine (PS), are monovalent and participate in cell signalling by helping to recruit signalling proteins to the plasma membrane through electrostatic interactions [8]. Polyvalent lipids such as phosphoinositides (PIs) also exist, albeit at lower abundance. For example phosphatidylinositol (4,5)-bisphosphate (PI(4,5)P_2_) typically makes up around 1% of the lipids in the cytoplasmic leaflet of the plasma membrane [5]. Though comparatively rare, PIs are involved in the regulation of several cell signalling pathways. From the PI framework structure, it is possible to generate seven physiological phosphatidylinositol phosphates (PIPs), which are differentiated by the number of substituent phosphate groups and pattern of phosphorylation around the inositol ring. The net negative charge is dependent on the phosphorylation motif [9], and so each PIP can act as a distinct target for a given class of proteins. The well-defined distribution of PIP molecules between the cytosolic membranes aids spatial regulation of protein recruitment. For example PI(4,5)P_2_ and phosphatidylinositol (3,4,5)-trisphosphate (PI(3,4,5)P_3_) are predominantly found in the plasma membrane, whereas PI(4)P is mainly restricted to the Golgi apparatus [10].

**Figure 1:**
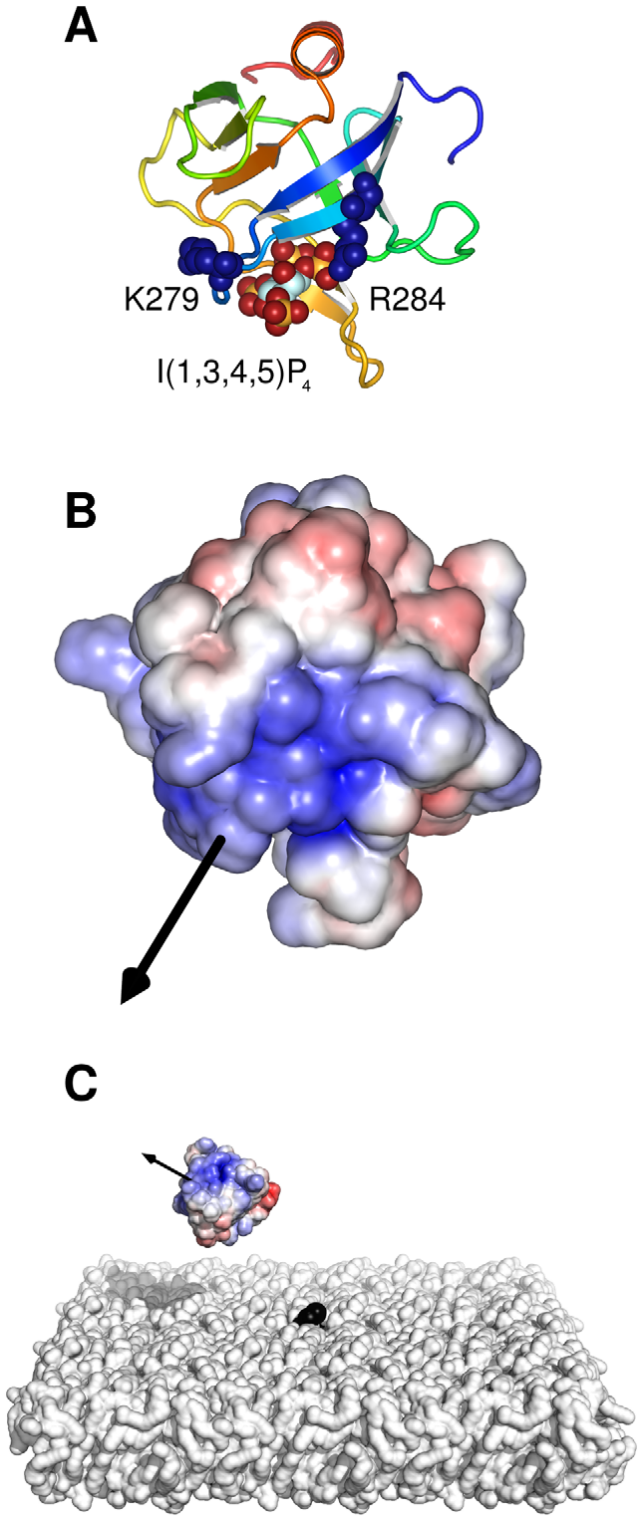
Structure of the GRP1 PH domain. **A** The crystal structure of GRP1-PH (PDB 1FGY). The I(1,3,4,5)P_4_ headgroup and the sidechains of two key residues (K279 and R284) are shown as van der Waals spheres. The protein is oriented such that the bilayer normal, as determined by previous MD simulations, is vertical (*i.e.* defines the *z* axis). **B** Electrostatic potential of GRP1-PH projected onto the solvent-accessible surface, showing the large positive electrostatic potential around the I(1,3,4,5)P_4_ binding site. The electrostatic potential was calculated in the absence of I(1,3,4,5)P_4_ using APBS as described in the main text and is coloured from −5 kT/*e* (red) to +5 kT/*e* (blue). The protein is shown in the same orientation as in **A** and the molecular dipole moment (calculated using the Protein Dipole Moments Server (http://bioinfo.weizmann.ac.il/dipol/; [70]) is indicated by a black arrow. **C** Snapshot from a BD simulation showing the protein solvent-accessible surface coloured by electrostatic potential, with the molecular dipole moment shown as a black arrow as in **B**. The POPC lipid bilayer is shown as a white surface with the single PI(3,4,5)P_3_ molecule shown in black.

A number of protein domains are involved in membrane recognition by signalling and trafficking proteins [1], [10], with the pleckstrin homology (PH) domain one of the most widespread. This is a structurally conserved domain of approximately 100 amino acid residues [11], [12] which is found in many signalling proteins, and in many cases is thought to play a role in targeting proteins to the surface of the plasma membrane by recognising specific phospholipids, in particular the PIPs [1], [13]. One well studied example of this family is the PH domain within the general receptor for phosphoinositides isoform 1 (GRP1; Figure 1). GRP1 is a member of the cytohesin family of proteins, and is responsible for catalysing GDP/GTP exchange on ADP-ribosylation factor (ARF) GTPases at the membrane surface. The anionic lipid PI(3,4,5)P_3_ acts to recruit GRP1 to the plasma membrane through electrostatic interactions, and the GRP1 PH domain reversibly binds PI(3,4,5)P_3_ with high affinity [14].

The importance of electrostatic interactions in binding of GRP1-PH [15] and related PH domains [16] to membranes has been demonstrated. It has been suggested that GRP1-PH first interacts with the membrane *via* weak nonspecific interactions with background anionic lipids, thus increasing the residence time at the membrane surface, and facilitating subsequent two dimensional diffusion to allow the protein to locate its target PI(3,4,5)P_3_ molecule [15]. It is therefore of interest to explore electrostatic steering of the GRP1 PH domain to the inner leaflet of the plasma membrane not only by interactions between the target PIP lipid and the PH domain, but also between the more general anionic lipid background and the PH domain. In particular, we wish to know to what extent the anionic background aids steering as opposed to acting as a ‘decoy’ luring the PH domain away from its target PIP molecule. This is a specific example of a more general problem of encounter and recognition within the crowded environment of the interior of the cell [17], [18], [19], [20].

Such problems may be addressed by computer simulation methods, including Brownian dynamics (BD) simulations which have previously been used to model processes ranging from enzyme-substrate encounters [21] to protein folding within the crowded environment presented by bacterial cytoplasm [22]. BD simulations have been used extensively to model protein-protein encounters in aqueous solution [23], and also those involving membrane proteins [24], [25], [26]. This suggests BD simulations are well suited to explore long range interactions governing PH/PIP encounters. Models of protein-protein association in solution typically incorporate two distinct steps. The diffusing partner molecules first interact through electrostatic interactions over a long range, approaching closely and then forming an initial encounter complex. The second step involves the relaxation and conformational rearrangement of the two partners within the encounter complex to form the final bound complex [27]. Thus one might anticipate protein-membrane interactions to also involve two or more comparable stages.

Guided by these considerations, we have conducted a multiscale simulation study in which we employ BD to model the initial encounter between the protein and the membrane, subsequently switching to atomistic molecular dynamics (MD) simulations to model the formation of the membrane-bound PH-PIP complex. Using this approach we demonstrate electrostatic competition between target (PIP) and decoy (anionic) lipids for the PH domain. Significantly, we show that the experimentally observed lipid composition of the cytoplasmic leaflet is optimal for electrostatic steering of the PH domain to the PIP target.

## Results

### Brownian Dynamics Simulations

**Figure 2:**
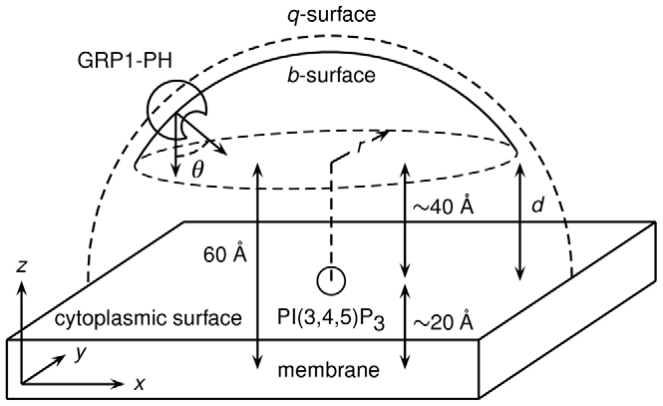
Schematic diagram of the BD simulation setup. The *b*-sphere is truncated to form an open hemispherical surface situated on one side of the membrane, with the value of *q* set such that all trajectories are terminated before the protein is able to diffuse outside the perimeter of the membrane patch. The three coordinates *r*, *z* and *θ* specify the position and orientation of the protein relative to the PI(3,4,5)P_3_ headgroup.

All simulations started with the protein randomly positioned and oriented relative to a lipid bilayer membrane. The protein centre lay on a hemispherical open surface of radius 100 Å and with *z*>60 Å, ensuring that the protein was always at least *d* = 40 Å away from and perpendicular to the cytoplasmic surface of the bilayer at the start of the simulation (see Methods and Figure 2). We carried out an ensemble of 5000 BD simulations for each system (Figure 1C shows a snapshot from one of these BD simulations). We then examined the distributions of the position and orientation of the PH domain relative to the target PI(3,4,5)P_3_ headgroup as specified by the three coordinates *r*, *d* and *θ* (Figure 2), evaluated across the time courses of these ensembles of simulations. We also recorded the coordinates of the PH domain (*r*, *d*, *θ*) upon first encounter of the protein and bilayer for each simulation.

Our model for a PI(3,4,5)P_3_-containing membrane was taken from previous atomistic MD simulations [28], replicated in the *x*,*y*-plane to generate a square bilayer patch of approximately 1560 POPC lipids with a single PI(3,4,5)P_3_ molecule in its centre. The final dimensions of the bilayer were approximately 220 Å×220 Å×40 Å.

**Figure 3:**
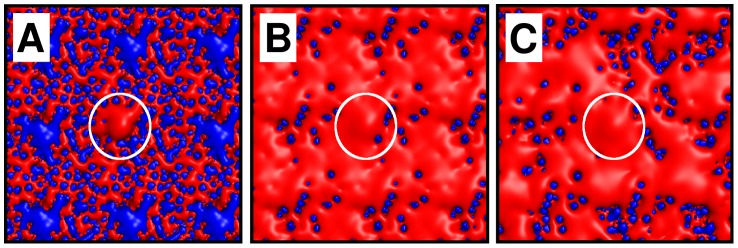
Lipid bilayer models. **A** Electrostatic potential isocontours of a POPC lipid bilayer (red = negative, blue = positive), with a central PI(3,4,5)P_3_ molecule clearly visible as a region of negative electrostatic potential (indicated by a white circle). **B** Electrostatic potential isocontours after modifying lipids with an evenly distributed fractional charge. Each lipid headgroup of the upper (*i.e.* cytoplasmic) leaflet of the bilayer has a fractional charge of −0.4 *e*. **C** Isocontours after a randomly selected subset of 40% of the lipids in the upper leaflet were assigned a charge of −1.0 *e*.

In the first instance, we performed BD simulations with an uncharged, zwitterionic POPC membrane, with only the PI(3,4,5)P_3_ molecule carrying a net negative charge (Figure 3A). To mimic the presence of anionic lipids in BD simulations of charged membranes, we assigned negative charges to the phosphatidylcholine lipid headgroup. Initially, we assigned identical, fractional negative charges to all of the nitrogen atoms to generate an even charge distribution across the surface (Figure 3B). In this case, the diffusing protein effectively interacts with the average charge distribution of the lipid bilayer.

The topography of the electrostatic potential at the membrane surface is dependent upon the distribution of the lipids. The lipid bilayer is dynamic and lipids undergo two-dimensional lateral diffusion in the plane of the membrane, and so the charge distribution is likely to fluctuate over time. The lateral diffusion constant of POPC lipids at 300 K is 1.7×10^−8^ cm^2^ s^−1^ [29]. However, the calculated diffusion constant of GRP1-PH at 300 K is 1.0×10^−6^ cm^2^ s^−1^ (see Methods), almost two orders of magnitude larger. Although care must be taken when attempting to directly compare two- and three-dimensional diffusion constants (see *e.g.* [30], [31]), this indicates that the protein may be more mobile than the lipids, with timescales of approximately 40 ns and 30 µs respectively for a 50 Å diffusional motion. This suggests that we should examine further the consequences of assuming the interaction of the protein with the average charge distribution of the lipid bilayer.

To explore this, we also applied an alternative approach in which we assigned a single negative charge to subset of the headgroups selected randomly (Figure 3C), thus generating a less even distribution of decoy negative charges. In this case, the protein interacts with a discrete distribution of charges, which is more in keeping with faster diffusion of the protein relative to the lipids. The fractional charges explored in the first set of simulations ranged from −0.2 *e* to −1.0 *e*; the number of lipids randomly assigned a charge of −1.0 *e* ranged from 20% to 100% in the second approach.

Another alternative method for exploring slower lipid diffusion is to generate lipid configurations *via* coarse grained molecular dynamics (CGMD) simulations of a lipid bilayer and then extract representative snapshots for use in the BD simulations. This has an advantage over the simple random assignment described above in that it is able to account for more complex lipid bilayer phenomena such as lipid demixing.

### Positional Steering

Initially, we were interested to see how easily GRP1-PH could locate its target lipid PI(3,4,5)P_3_ when the surrounding membrane contained increasing numbers of negatively charged lipids. It might be expected that increasing the (negative) surface charge density would disrupt GRP1-PH targeting by masking the position of the negatively charged PI(3,4,5)P_3_.

**Figure 4:**
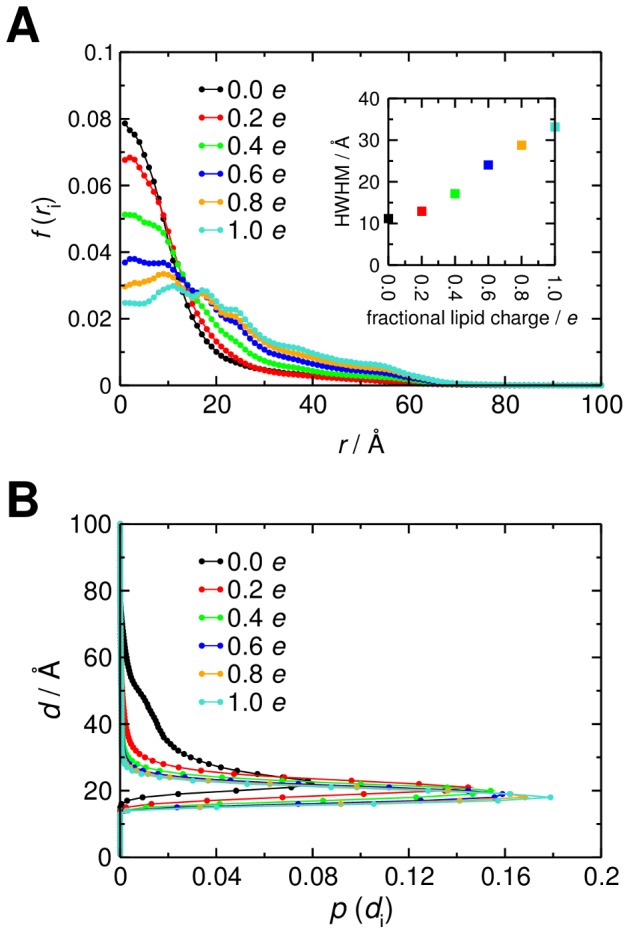
Positional steering of GRP1-PH for the initial bilayer model. **A** Distribution of radial locations, *r*, of GRP1-PH over the course of the 5000 BD simulations in each ensemble for the case of evenly distributed, fractional charges ranging from 0.0 *e* (no anionic lipids) to 1.0 *e* (all anionic lipids in the cytoplasmic leaflet of the bilayer). The centre of mass of the ‘target’ PI(3,4,5)P_3_ headgroup is located at *r* = 0. The inset graph shows the half width at half maximum (HWHM) for each of the *r* distributions, calculated by fitting a single Gaussian centred at *r* = 0 to each data set. The HWHM is plotted against the fractional negative charge assigned to the lipid headgroups. **B** Distribution of *d* positions of the protein for the same set of even charge distribution BD simulations. The centre of mass of the PI(3,4,5)P_3_ headgroup, defining the surface of the bilayer, lies at *d* = 0.

With only PI(3,4,5)P_3_ present in a POPC bilayer (0.0 *e*) GRP1-PH spends the majority of the trajectory diffusing close to its target lipid, evidenced by the large peak at small values of *r* (Figure 4A). However, when the surface charge is increased (−0.2 to −1.0 *e*) the peak height diminishes and the maximum shifts to larger values of *r*, which appears to suggest a reduction in positional steering with the protein much less likely to closely approach the PI(3,4,5)P_3_ molecule. To assess the degree of targeting, we extracted the peak half-width at half maximum (Figure 4A, inset), with low values corresponding to narrow distributions of *r* and efficient PI(3,4,5)P_3_ positional steering, while higher values correspond to wide distributions of *r* and comparatively poor PI(3,4,5)P_3_ positional steering. It is also of interest to investigate how the distribution of *d* positions (*i.e.* along the bilayer normal) of the protein changes depending upon the membrane charge. As anticipated, as the membrane negative charge increases, GRP1-PH spends more time closer to the surface of the bilayer (Figure 4B). It is noteworthy that a −0.2 *e* surface charge on the cytoplasmic leaflet (corresponding to an overall bilayer composition of 10% anionic lipids, close to that observed experimentally [7]) results in the PH domain spending the majority of its time close to or at the bilayer surface (smaller values of *d* along *z*) without significant diffusion away from (on *r*) the target PI(3,4,5)P_3_ molecule.

**Figure 5:**
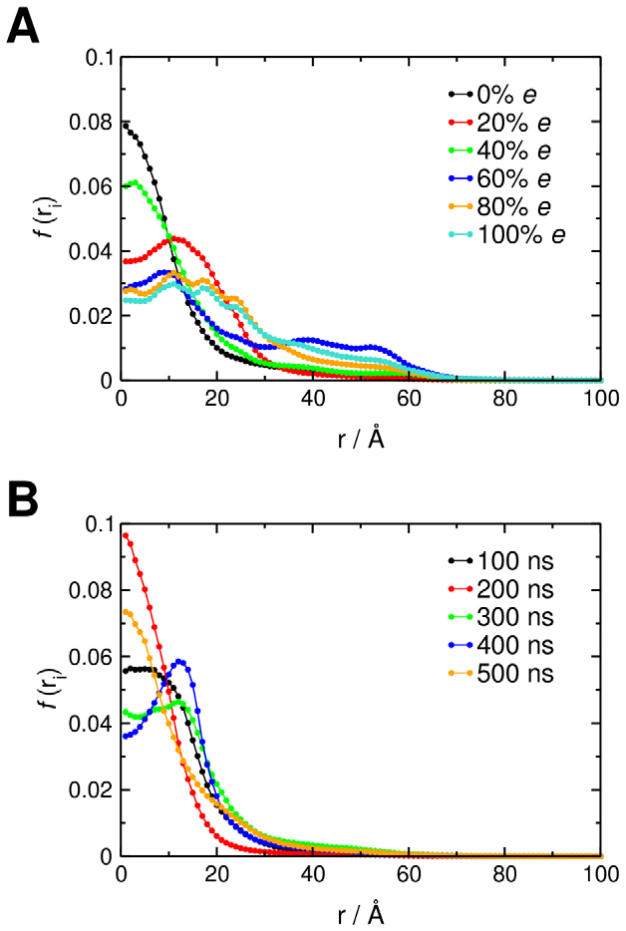
Positional steering of GRP1-PH using alternative lipid configurations. **A** Distribution of radial locations, *r*, for the BD simulations based on randomly distributed, integer-valued negative charges on the lipid headgroups. The centre of mass of the ‘target’ PI(3,4,5)P_3_ headgroup is located at *r* = 0. **B** Distribution of radial locations, *r*, for the BD simulations based on the lipid configurations generated from a CGMD simulation of a lipid bilayer containing 20% anionic lipids.

We repeated the simulations with integer negative charges located on individual lipids rather than fractional charges evenly distributed across all lipid headgroups. Initially, the lipids carrying negative charges were selected randomly (Figure 5A). In these simulations the same overall trends were observed but with some variations reflecting the fixed ‘snapshot’ of the mixed lipid bilayer used in the BD simulations. Thus the exact extent of the positional steering behaviour observed is dependent upon the instantaneous distribution of monovalent negatively charged lipids used in the BD simulation setup. We therefore speculate that lipid clustering might modulate positional steering of PH domains to PI(3,4,5)P_3_.

**Figure 6:**
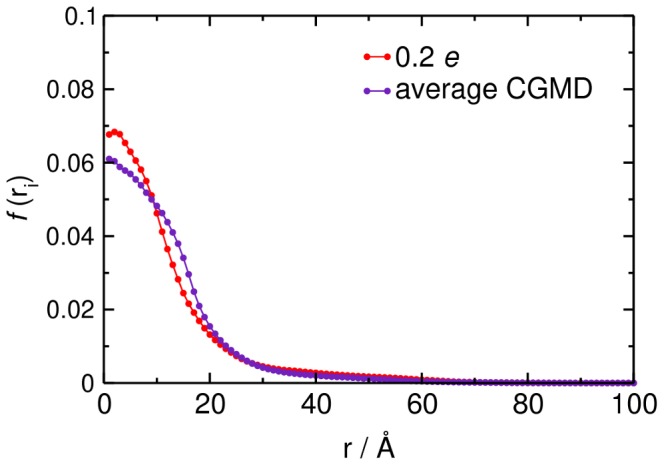
Comparing positional steering for different lipid configurations. Comparison of the profile generated by averaging the five *r* distributions (Figure 5B) from the BD simulations using the lipid configurations produced from a CGMD simulation with a 20% anionic lipid concentration (purple) with the *r* profile from the BD simulations using evenly distributed fractional charges of −0.2 *e* across all lipids (red).

To test this, we performed a 0.5 µs CGMD simulation of a mixed lipid bilayer containing 20% anionic lipids (see Methods) and extracted configurations at intervals of 100 ns (Figure S1). These five configurations were then used as an input for a set of BD simulations, to probe the sensitivity of GRP1-PH targeting to the distribution of anionic lipids. Despite the fact that the anionic lipid concentration is the same in each snapshot, we see variations in the positional steering for the different lipid configurations (Figure 5B), again suggesting that steering is influenced not only by the concentration of anionic lipids but also by the distribution of these lipids over the surface. Interestingly, if we take the average of the *r* distributions over these five sets of BD simulations, we generate a profile similar to that observed for the single set of BD simulations using the fractional charge distribution where each lipid is assigned a charge of −0.2 *e* (Figure 6). This suggests that the fractional charge distribution is a reasonably good model of the time-averaged behaviour of the system, allowing for lipid dynamics on the sub-microsecond timescale.

### Orientational Steering

As well as the effect of bilayer surface charge on the positional steering of GRP1-PH to the membrane, we also wished to investigate how surface charge might influence orientational steering of the protein as it approaches the surface, as this may be anticipated to influence the formation of a ‘productive’ GRP1-PH/PI(3,4,5)P_3_ complex upon encounter. GRP1-PH carries a dipole moment, and the vector is directed towards the binding cavity of the protein (Figure 1). In order to monitor the orientation of the protein over the BD trajectories, we calculated the angle, *θ*, made by a vector from the PH domain to the target PI(3,4,5)P_3_ and the *z* axis (see Supporting Information), with *θ* = 0° corresponding to the protein orientation seen in the docked GRP1-PH/PI(3,4,5)P_3_ complex observed in previous structural and MD simulation studies [28].

**Figure 7:**
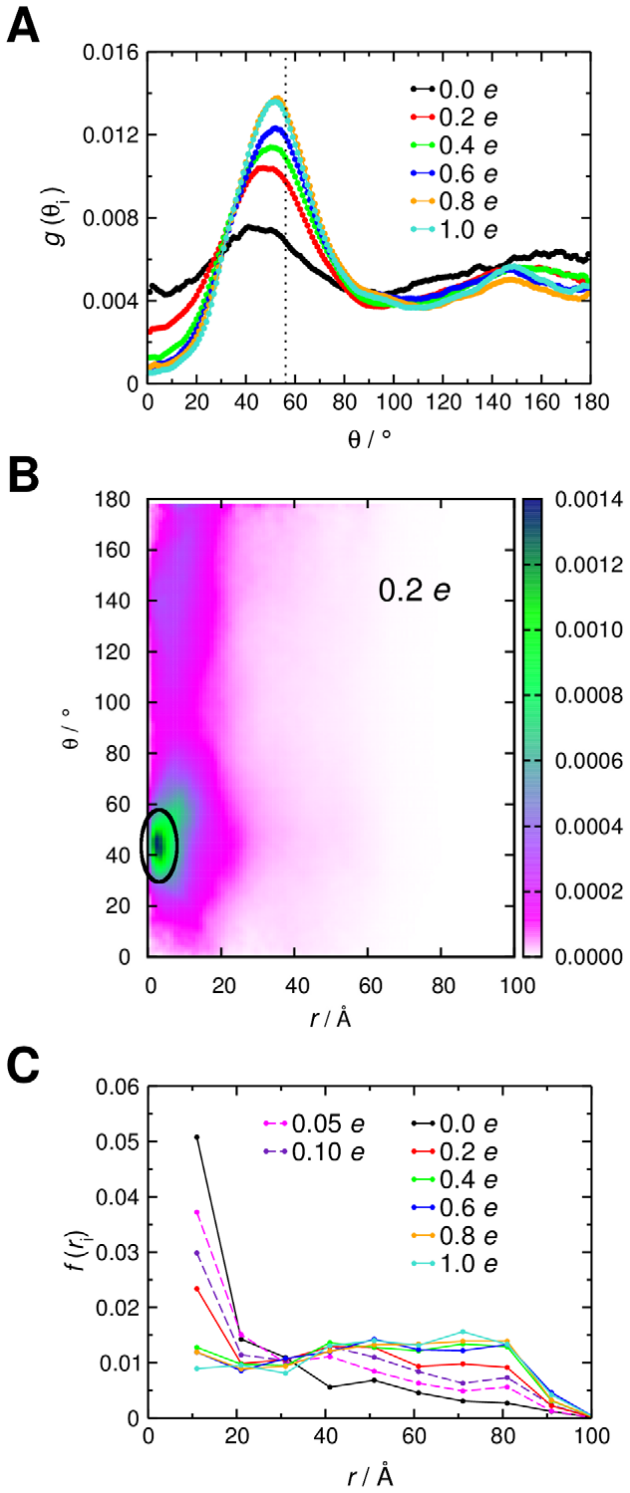
Orientational steering of GRP1-PH. **A** Distribution of orientations, *θ*, of GRP1-PH over the course of the BD simulations for the case of the evenly spread, fractional charges from 0.0 to −1.0 *e*. The dotted line at *θ* = 56° corresponds to the angle, *θ*, that the molecular dipole moment makes with respect to the *z* axis in the membrane bound complex. **B** Two-dimensional distributions of *θ vs. r* from the BD simulations with the fractional charge distributions with all lipids (other than PI(3,4,5)P_3_) given a charge of −0.2 *e*. Note that the origin corresponds to the configuration obtaining by manual docking plus simulations (see main text and [28]). **C** Distribution of radial ‘first encounter’ locations for the BD simulations with evenly spread, fractional charges from 0.0 to −1.0 *e*.

The distribution of *θ* as a function of negative surface charge shows a clear effect of a surface charge of −0.2 *e* or more on orienting the PH domain relative to the bilayer (Figure 7A). Thus the distribution of orientations shifts towards values of θ corresponding to alignment of the GRP1-PH dipole moment (which lies at an angle of 56° to the reference vector) with the membrane normal (Figure 7). The membrane charge therefore appears to influence not only the position of the protein but also its orientation

Although increasing surface charge aids orientational steering of GRP1-PH, this seems to come at the cost of some loss of positional steering. It seems that a level of negative surface charge (−0.2 *e*) close to that reported [7] in the cytoplasmic leaflet of mammalian plasma membranes may be optimal in achieving both forms of steering, as can be seen in a two-dimensional distribution of *r* and *θ* values adopted during a simulation with a −0.2 *e* bilayer (Figure 5B). To explore this further we analysed the distribution of first-encounter positions between the bilayer and the PH domain (Figure 7C). At −0.2 *e* there was clear positional steering of the PH domain to the PI(3,4,5)P_3_ molecule. Increasing the surface charge to −0.4 *e* or more resulted in almost complete loss of positional steering.

We performed BD simulations of two mutants of GRP1-PH (R284A and K279A) which have previously been shown to reduce binding of the protein to *soluble* inositol phosphates [32]. It was therefore of interest to see whether these mutations also influenced steering of the PH domain to PI(3,4,5)P_3_ in a lipid bilayer. The mutant R284A has been shown experimentally to almost completely abolish binding in solution whereas the K279A mutation has a smaller effect. Both mutants show a modest reduction in positional steering (Figure S2), and in the fraction of time spent close to the bilayer, with smaller values of *d* along *z*. This effect was more pronounced in the case of the R284A mutant, which correlates with the experimental results. This reduction in steering could be a contributory factor to the experimentally observed lower binding affinity, but clearly other effects such as conformational changes and sidechain-specific protein-lipid interactions may also be important.

### Molecular Dynamics Simulations

**Figure 8:**
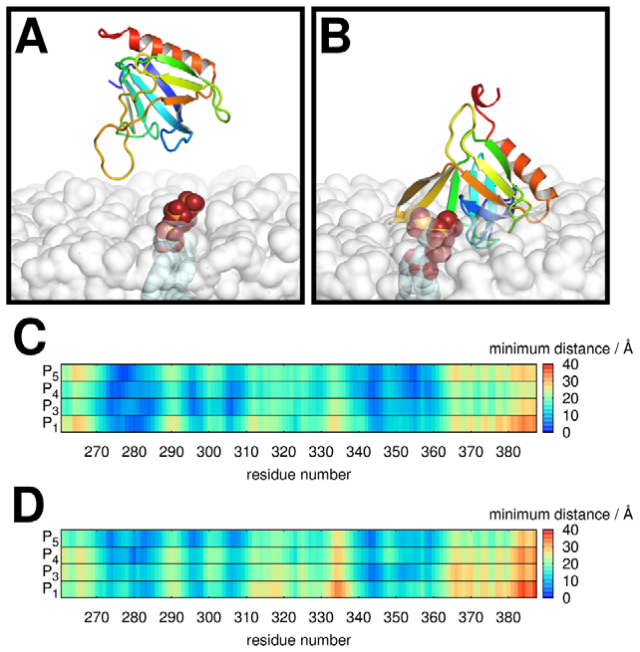
MD simulations of bound complex formation. **A** An optimal configuration for subsequent membrane-binding extracted from the ensemble of BD simulations conducted using an uncharged, zwitterionic lipid bilayer containing PI(3,4,5)P_3_. In this case *r* = 9 Å, *d* = 18 Å and *θ* = 27°. **B** A snapshot at the end of a subsequent 100 ns MD simulation. **C, D** Fingerprint plots showing the location of PH domain residues contacting the phosphorus atoms of PI(3,4,5)P_3_ in **C** the X-ray structure (PDB 1FGY) and **D** the 100 ns snapshot from the MD simulation shown in **B**. In each plot the minimum distances between each residue and each phosphorus atom are shown.

In order to implement a multiscale approach to simulating membrane binding of PH domains we combined BD simulations with subsequent MD simulations. Similar combined approaches have been successful in studying DNA-enzyme interactions [33]. Optimal encounter complexes from the BD simulations, *i.e.* in which both positional and orientational steering were observed, were used as initial configurations for atomistic MD simulations to explore the conformational changes involved in complex formation. As seen above a suitable configuration for binding is likely to be one with small values of *r*, *d* and *θ* simultaneously, with GRP1-PH in close proximity to PI(3,4,5)P_3_ with its binding cavity and also its dipole moment oriented towards the ligand. We therefore performed a simple search of the trajectories in order to locate a configuration satisfying these requirements. One such optimal configuration (*r* = 9 Å, *d* = 18 Å, *θ* = 27°) was extracted and we performed two MD simulations to test whether the protein was able to bind to its target lipid from this position (Figure 8). In both MD simulations GRP1-PH approaches the PI(3,4,5)P_3_ molecule in the lipid bilayer, with the separation between the centre of mass of the protein and that of the IP(1,3,4,5)P_4_ headgroup falling to 15 Å in both cases. This is in good agreement with the centre-to-centre separation of 13 Å found in the ligand-bound crystal structure (PDB 1FGY [34]). To investigate the geometry of the complex, we mapped the minimum distance between each amino acid residue of the protein and each of the phosphorus atoms of the I(1,3,4,5)P_4_ headgroup in the crystal structure. This revealed a characteristic protein-ligand interaction ‘fingerprint’ which agreed well with that seen in both of the simulations (Figure 8). After a 100 ns MD simulation the protein locates the membrane-bound PI(3,4,5)P_3_ within the first 20 ns of simulation, and binds *via* a set of amino acid residues similar to that found in the crystal structure. This set of interactions is preserved throughout the 100 ns simulation.

## Discussion

We have used a multiscale simulation approach, combining BD and MD simulations, to characterise in atomic detail the association of GRP1-PH with a PI(3,4,5)P_3_-containing lipid bilayer. The BD simulations reveal how long range electrostatic interactions steer the PH domain, both positionally and orientationally, towards the PI(3,4,5)P_3_-containing anionic bilayer surface. There appears to be a payoff between non-PI(3,4,5)P_3_ anionic lipids attracting the PH domain to the bilayer surface in a favourable orientation, and their acting as ‘decoys’ for interaction of PH with the PI(3,4,5)P_3_ molecule. This provides a refinement of an earlier model of the role of background anionic lipids in PH domain binding [15]. It is notable that the dipole moment vector of GRP1-PH points from the centre of mass of the protein towards the location of the bound PI headgroup. This, coupled with the observation that increasing surface charge leads to enhanced alignment of the dipole moment with the membrane normal, suggests that the orientation of the molecular dipole moment may play an important role in successful PH domain targeting. Evaluation of the molecular dipole moments for a variety of other PH domains suggests that this orientation is a conserved feature of PH domains (Figure S3). Thus, it is likely that the GRP1-PH targeting behaviour observed here may be conserved across the PH domain family.

Significantly, a typical level of anionic lipid in the bilayer (approximately 20% in the cytoplasmic leaflet) seems to be optimal to both enhance orientational steering and to localise GRP1-PH proximal to the surface of the bilayer without sacrificing its ability to locate PI(3,4,5)P_3_ within the bilayer plane. Thus the protein is steered into the correct orientation for binding by the higher surface charge density as compared with a zwitterionic membrane, but the charge density is not yet high enough to mask the position of PI(3,4,5)P_3_, thereby allowing for efficient positional steering. Finally, we demonstrate that using appropriate encounter complexes from the BD simulations as initial configurations for atomistically detailed MD simulations, which include explicit solvent molecules and intramolecular motions, leads to formation of a GRP1-PI(3,4,5)P_3_ complex at the membrane surface that accurately reproduces the geometry of the bound complex from the crystal structure. This combined BD-MD technique therefore provides a means to model the membrane binding modes of lipid-recognition proteins, a class of proteins which play a number of key roles in membrane function [1] and disease [35]. With respect to PH/PI(3,4,5)P_3_ recognition we arrive at an overall model which combines electrostatic steering directly to the target PI(3,4,5)P_3_, possibly with an element of non-specific (electrostatic) bilayer association, followed by 2D diffusion at the surface until the PH/PI(3,4,5)P_3_ encounter occurs. This process is likely to involve further complexities related to multiple membrane targeting domains binding to more than one target lipid [36].

Our findings contribute to a more general consideration of lipid bilayer composition and recognition by protein domains (see *e.g.* [1], [5], [37]). The test system used in this study, with a PI(3,4,5)P_3_/lipid ratio of approximately 1∶1000 is likely to be (globally) representative of mammalian cell plasma membranes. While it is difficult to estimate the physiological concentration of PI(3,4,5)P_3_, which varies according to the level of cell stimulation, PI(3,4,5)P_3_ is generated from PI(4,5)P_2_, the concentration of which is around 1% of cell membrane lipids [5]. Thus, if we assume that even at the peak of cell stimulation the concentration of PI(3,4,5)P_3_ will be less than 1%, then the PI(3,4,5)P_3_ concentration present in our simulations is of the correct order of magnitude. Of course, this is something of a simplification given the importance of localisation and gradients of PI(3,4,5)P_3_ in cell signalling and dynamics [38] and also possible larger scale differences in PIP composition in plasma membranes between the apical and basal regions (with higher concentrations of PI(4,5)P_2_ and PI(3,4,5)P_3_ respectively) in epithelial cells [39]. These studies indicate that it is essential to the function of a cell that domains such as GRP1-PH not only bind in a stable fashion to their cognate PIPs but are able to locate them in complex ‘mixed’ systems similar to those present *in vivo*.

It is important to consider the limitations of the current model. Our BD simulations treat the bilayer as a static entity lacking any internal dynamics. This is likely to be sufficient to capture longer range steering interactions, but a more dynamic model may be needed if this approach is to be applied to larger, more complex membrane systems. One option would be to combine BD for longer range protein/membrane interactions with a CGMD [40] approach to generate and update configurations of a mixed lipid bilayer. In particular such an approach should enable one to capture effects whereby cationic proteins interacting with a membrane surface may result in redistribution of anionic lipids within the membrane [41], [42], [43], [44]. It has been suggested that this can result in correlated diffusion of lipids and protein [45] and in enhancement of the binding affinity of a protein by charged lipids having a higher local concentration in its vicinity [46], [47]. The BD simulations presented here also neglect any effect of hydrodynamic interactions on the association process [48], [49], [50], [51]. This has been suggested to lead to potential problems in simulations of protein/protein association in solution, and should be explored for any effects on a protein diffusing close to a membrane surface.

In our analysis of the results of the BD simulations we distinguish between positional and orientational aspects of electrostatic steering. We make this distinction as it is necessary for both types of steering (positional and orientational) combine favourably to yield a ‘productive’ encounter complex. In contrast, one could imagine a scenario whereby the PH domain closely approaches the PI(3,4,5)P_3_ ligand (*i.e.* good positional steering) but with its molecular dipole in the wrong orientation for binding (*i.e.* poor orientational steering).

From a biological perspective the main limitation is that our simulations mimic *in vitro* biophysical studies, with a simplified bilayer lipid composition. Current lipidomics studies [7] are revealing the spatial and temporal complexities of membrane lipid composition within living cells. Furthermore, recent studies of syntaxin-1A/PIP_2_ interactions [52] indicate that electrostatic interactions between PIP_2_ and the basic residues in the juxtamembrane region of syntaxin-1A result in formation of approximately 75 nm diameter PIP_2_-rich microdomains in the inner leaflet of PC12 cell plasma membranes. Therefore, it seems likely that our studies have only scratched the surface in terms of understanding how the GRP1-PH domain locates and binds to a PI(3,4,5)P_3_ molecule within a cell membrane. However, by combining previous approaches using electrostatics calculations [5] and detailed MD simulations [28], they provide a computational framework to enable us to begin to address the more complex cell membrane environment, thus offering a link from membrane protein structure and biophysics through to cell biology of membranes.

## Methods

### Lipid Bilayer Model

Our model for a PI(3,4,5)P_3_-containing phospholipid bilayer was taken from previous MD simulations [28], replicated in the *x* and *y* directions to generate a square bilayer patch comprising approximately 1560 POPC lipids with a single PI(3,4,5)P_3_ molecule in the centre. The final dimensions of the bilayer were approximately 220 Å×220 Å×40 Å. Atomic partial charges on the lipids were identical to those used previously [28]. In the first instance, we carried out BD simulations with an uncharged, zwitterionic POPC membrane, with only the PI(3,4,5)P_3_ molecule carrying a net negative charge. To mimic the presence of anionic lipids in BD simulations of charged membranes, we assigned negative charges to the nitrogen atoms of the phosphatidylcholine headgroups of the POPC lipids. As discussed above we either assigned identical, fractional negative charges to all of the nitrogen atoms to generate a relatively even charge distribution across the surface or assigned a single negative charge to a random subset of the nitrogen atoms, to generate an uneven distribution of negative charge.

### Electrostatics Calculations

Finite difference Poisson-Boltzmann calculations were carried out using the APBS software [53]. The Poisson-Boltzmann equation was solved at a temperature of 300 K and an ionic strength of 0.1 M using cubic grids of dimensions 385×385×385 for the bilayer and 129×129×129 for the protein, each with a 1 Å spacing. Grids were centred on the centre of mass of the bilayer and of the protein respectively.

### Brownian Dynamics Simulations

Brownian dynamics simulations were performed using SDA version 5.01 [54]. While the specifics of the SDA software are documented in detail elsewhere, for completeness we briefly review the method here. The diffusion equation is solved using the algorithm developed by Ermak and McCammon [55], and the translational Brownian motion of the protein is simulated as the displacement Δ**r** of the relative separation vector **r** during a time step Δ*t* according to the relation:
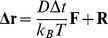
where **F** is the force on the protein, **R** is a random vector that satisfies 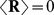 and 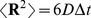 and the prefactor *D/k_B_T* represents the solvent friction. Rotational motions are treated in an analogous fashion:
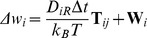
where **T** *_ij_* is the torque on the protein and **W** is a random rotation angle that satisfies
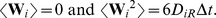
The forces between the diffusing protein and the target bilayer are computed as finite-difference derivatives of the free energy of interaction between the protein and the bilayer.
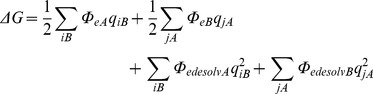
The unfavourable electrostatic desolvation term is given by:
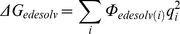
This is approximated by:
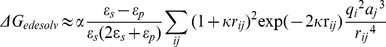
As in previous studies, the scaling factor α was set to 1.67.

Electrostatic desolvation grids were calculated according to the protocol developed by Elcock *et al.* [56]. HYDROPRO [57] was used to estimate the translational and rotational diffusion constants for the protein giving values of *D* = 1.042×10^−6^ cm^2^ s^−1^ and *D_R_* = 1.656×10^7^ rad s^−1^ respectively. We used the effective charge method [58] to assign partial charges to the protein.

We modified the SDA source code to truncate the *b*-sphere, such that all trajectories began on the hemispherical open surface given by *r* ^2^ = *x* ^2^+*y* ^2^+*z* ^2^, *z*>60 Å. As the width of one leaflet of the bilayer is around 20 Å, this ensured that the protein always lay at least *d* = 40 Å distant from the surface of the bilayer at the start of the trajectory (Figure 2). Rotational diffusion of the lipid bilayer was switched off. The Debye length of the system at an ionic strength of 0.1 M is approximately 10 Å, and accordingly we chose the radius of the *b*-surface to be 100 Å. Our bilayer patch was approximately 220 Å wide, and so we elected to set the *q*-surface to 105 Å. This resulted in the termination of any trajectory that came close to the edge of the bilayer, thus limiting edge effects. We carried out 5000 BD simulations for each system. We note that our truncation of the *b*-sphere coupled with our choice of the *q*-surface is likely to invalidate the Northrup-Allison-McCammon (NAM) method for computing reaction rates [59], since the ensemble reactive flux is no longer spherically symmetric. However the focus of this study was to investigate the behaviour of the protein as it explored the bilayer surface, rather than attempt to calculate reaction rates explicitly.

### Histogram Construction

One problem when attempting to plot histograms of *r = x^2^+y^2^* is that the bin width between *r* and *r + dr* is not constant, and so the area of the bin is proportional to *r*. This has the effect of overrepresenting the larger distances in the histogram. One way of remedying this is to reweight the data by some appropriate factor to compensate for this effect [60]. In plane polar coordinates the Jacobian factor is 2*πr*, and so we rescale the data in each of the bins of our histogram:
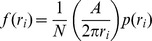
The same issue occurs when constructing histograms of the orientation of the protein, which can be represented by a unit vector rotating in space. Plotting histograms of the distribution of angles that this vector makes with the *z* axis is problematic since the areas of the spherical segments between *θ* and *θ + dθ* are not equal, which again distorts the distribution. In spherical polar coordinates the Jacobian factor is *r^2^*sin(*θ*), and so we rescale our histograms of the protein orientation using the following:
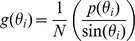



### Molecular Dynamics Simulations

MD simulations were carried out with GROMACS version 4.0.5 [61] using the GROMOS96 43a1 forcefield [62]. Simulations were run at 300 K with temperature kept constant by coupling to a Berendsen thermostat [63] with a coupling constant of τ_T_ = 0.1 ps. Pressure was maintained at 1 atm using a Parrinello-Rahman barostat [64], [65] and semi-isotropic pressure coupling, with τ_p_ = 1.0 ps and a compressibility of 4.6×10^−5^ bar^−1^. The SPC water model [66] was used, and the system was energy minimised for up to 1000 steps using the steepest descent algorithm prior to the production runs. Simulations were carried out using a timestep of Δ*t* = 2 fs, and bond lengths and angles were constrained using the LINCS algorithm [67]. Independent simulations were initiated from the same system configuration but with a different set of initial velocities. The neighbour list was updated every 10 steps and atomic positions were written every 10 ps. Electrostatic interactions were treated with the particle mesh Ewald (PME) approach [68] with a short-range direct space cut-off of 10 Å.

### Coarse-Grained Molecular Dynamics Simulations

CGMD simulations were carried out with the MARTINI forcefield [69], using a timestep of 10 fs. In this CG model, zwitterionic lipids such as POPC are approximated by a positively charged particle (choline), a negatively charged particle (phosphate), two polar particles (glycerol) and two acyl chains made up of four and five hydrophobic particles respectively. We denote these lipids as CG4/5. Negatively charged lipids such as POPS are treated in a similar fashion, except that the positively charged particle is now replaced with a polar particle to represent the switch from choline to serine. These negatively charged CG lipids are therefore denoted as CG4/5–. The CGMD simulation comprised a mixture of approximately 1560 lipids in total, with CG4/5∶CG4/5– lipids in a ratio of 80∶20 in the PI(3,4,5)P_3_-containing upper leaflet of the lipid bilayer and pure CG4/5 lipids in the lower leaflet of the lipid bilayer. No evidence of lipid flip-flop between bilayer leaflets for the CG4/5– lipids was observed over the simulation.

## Supporting Information

Figure S1 **Lipid bilayer models based on CGMD simulations.** Electrostatic potentials calculated from lipid distributions obtained from CGMD simulations of a bilayer containing 20% negatively charged lipids. Electrostatic potentials were calculated using snapshots taken from the simulation at **A** 100 ns, **B** 200 ns, **C** 300 ns, **D** 400 ns and **E** 500 ns.
(TIFF)

Figure S2 **Positional steering of mutant GRP1-PH.** **A** Distribution of positions, *r*, of the K279A and R284A mutants over the course of the BD simulations as compared to the wild type. **B** Distribution of *z* positions of the protein for the two mutants compared to the wild type.
(TIFF)

Figure S3 **Molecular dipole moments for a selection of structures of PI-binding PH domains.** **A** GRP1-PH (PDB 1FGY [1]); **B** PLC-δ1 (PDB 1MAI [2]); **C** DAPP1 (PDB 1FAO [3]); **D** BTK (PDB 1B55 [4]); **E** PKB/Akt (PDB 1H10 [5]). In each case the molecular dipole moment points approximately towards the location of the bound PI ligand, indicating that this may be a general structural feature of the PH domain family with implications for membrane targeting behaviour.
Lietzke SE, Bose S, Cronin T, Klarlund J, Chawla A, et al. (2000) Structural basis of 3-phosphoinositide recognition by pleckstrin homology domains. Mol Cell 6: 385–394.

Ferguson KM, Lemmon MA, Schlessinger J, Klarlund J, Sigler PB (1995) Structure of the high affinity complex of inositol trisphosphate with a phospholipase C pleckstrin homology domain. Mol Cell 83: 1037–1046.

Ferguson KM, Kavran JM, Sankaran VG, Fournier E, Isakoff SJ, et al. (2000) Structural basis for discrimination of 3-phosphoinositides by pleckstrin homology domains. Mol Cell 6: 373–384.
Baraldi E, Carugo KD, Hyv—nen M, Surdo PL, Riley AM, et al. (1999) Structure of the PH domain from Bruton’s tyrosine kinase in complex with inositol 1,3,4,5-tetrakisphosphate. Structure 7: 449–460.Thomas CC, Deak M, Alessi DR, van Aalten DMR (2002) High-resolution structure of the pleckstrin homology domain of protein kinase B/Akt bound to phosphatidylinositol (3,4,5)-trisphosphate. Current Biology 12: 1256–1262.
(TIFF)
