# Supplementary figures and images for: Finding a Needle in a Haystack: The Role of Electrostatics in Target Lipid Recognition by PH Domains
Source: PLoS Comput Biol. 2012 Jul 26;8(7):e1002617. doi: 10.1371/journal.pcbi.1002617 (PMC3406000; doi:10.1371/journal.pcbi.1002617)

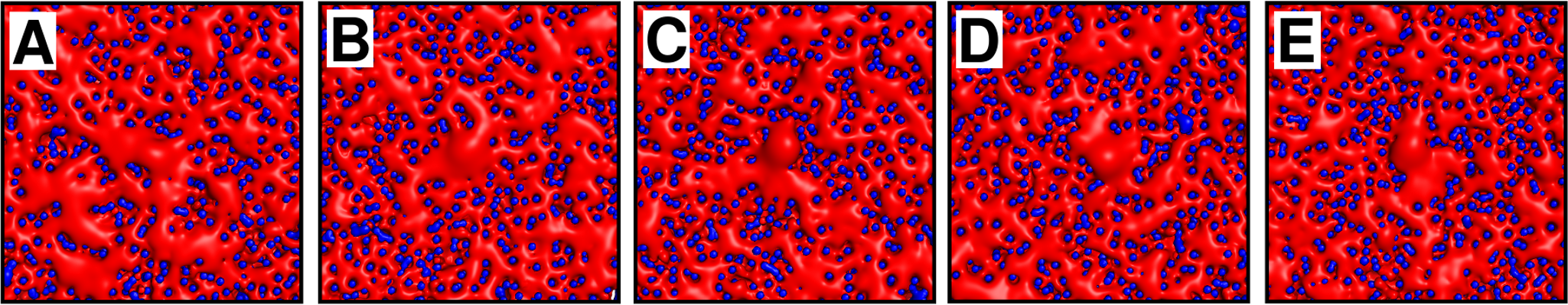

Supplement: Figure S1 — Lipid bilayer models based on CGMD simulations. Electrostatic potentials calculated from lipid distributions obtained from CGMD simulations of a bilayer containing 20% negatively charged lipids. Electrostatic potentials were calculated using snapshots taken from the simulation at A 100 ns, B 200 ns, C 300 ns, D 400 ns and E 500 ns. (TIFF) [file pcbi.1002617.s001.tiff]

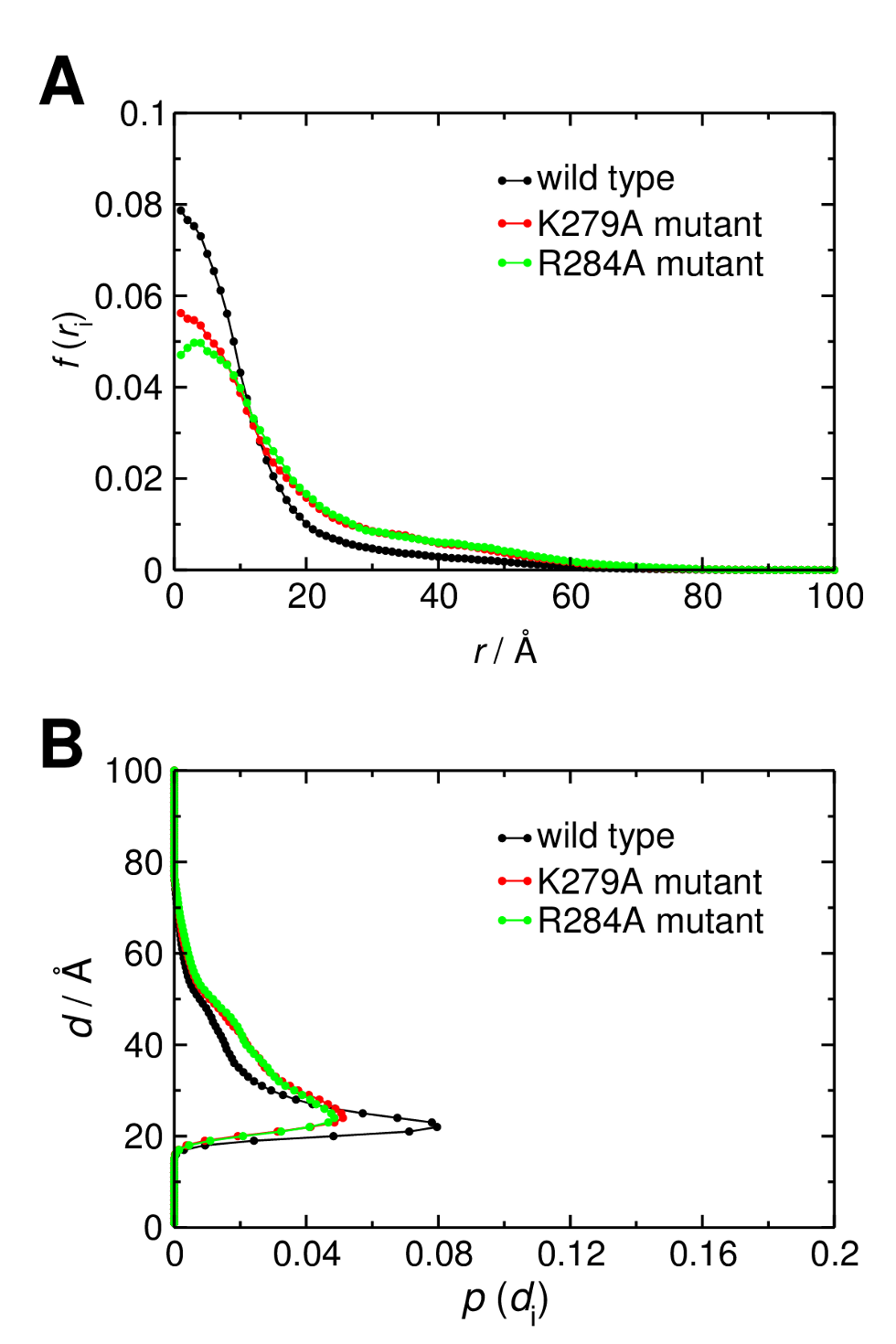

Supplement: Figure S2 — Positional steering of mutant GRP1-PH. A Distribution of positions, r, of the K279A and R284A mutants over the course of the BD simulations as compared to the wild type. B Distribution of z positions of the protein for the two mutants compared to the wild type. (TIFF) [file pcbi.1002617.s002.tiff]

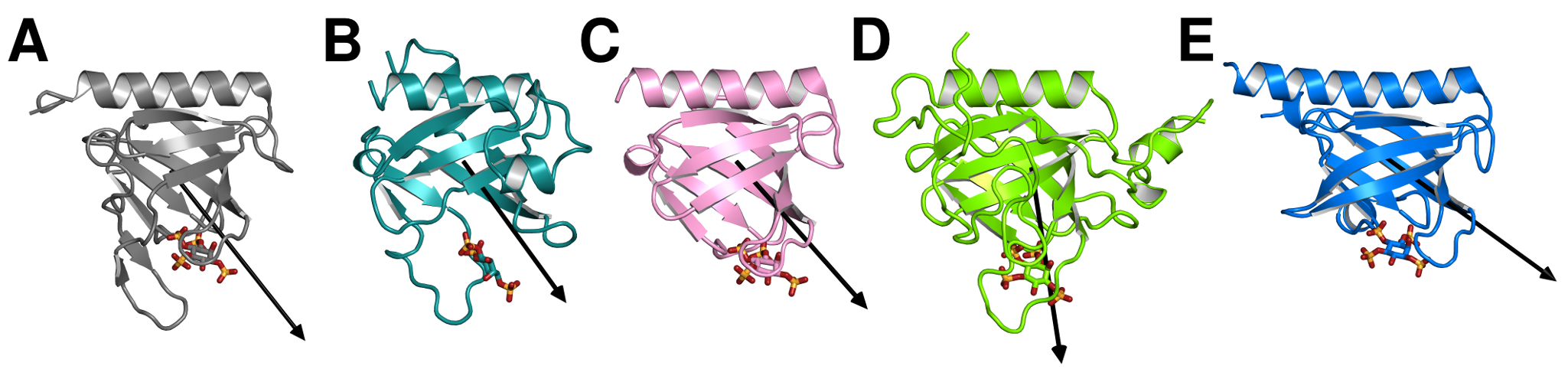

Supplement: Figure S3 — Molecular dipole moments for a selection of structures of PI-binding PH domains. A GRP1-PH (PDB 1FGY [1]); B PLC-δ1 (PDB 1MAI [2]); C DAPP1 (PDB 1FAO [3]); D BTK (PDB 1B55 [4]); E PKB/Akt (PDB 1H10 [5]). In each case the molecular dipole moment points approximately towards the location of the bound PI ligand, indicating that this may be a general structural feature of the PH domain family with implications for membrane targeting behaviour. Lietzke SE, Bose S, Cronin T, Klarlund J, Chawla A, et al. (2000) Structural basis of 3-phosphoinositide recognition by pleckstrin homology domains. Mol Cell 6: 385–394. Ferguson KM, Lemmon MA, Schlessinger J, Klarlund J, Sigler PB (1995) Structure of the high affinity complex of inositol trisphosphate with a phospholipase C pleckstrin homology domain. Mol Cell 83: 1037–1046. Ferguson KM, Kavran JM, Sankaran VG, Fournier E, Isakoff SJ, et al. (2000) Structural basis for discrimination of 3-phosphoinositides by pleckstrin homology domains. Mol Cell 6: 373–384. Baraldi E, Carugo KD, Hyv—nen M, Surdo PL, Riley AM, et al. (1999) Structure of the PH domain from Bruton’s tyrosine kinase in complex with inositol 1,3,4,5-tetrakisphosphate. Structure 7: 449–460.Thomas CC, Deak M, Alessi DR, van Aalten DMR (2002) High-resolution structure of the pleckstrin homology domain of protein kinase B/Akt bound to phosphatidylinositol (3,4,5)-trisphosphate. Current Biology 12: 1256–1262. (TIFF) [file pcbi.1002617.s003.tiff]
